# Neuroprotective, neurotherapeutic, and neurometabolic effects of carbon monoxide

**DOI:** 10.1186/2045-9912-2-32

**Published:** 2012-12-27

**Authors:** Vicki L Mahan

**Affiliations:** 1 St. Christopher’s Hospital for Children, Department of Pediatric Cardiothoracic Surgery, 3601 A Street, Philadelphia, PA, 19134, USA; 2 Drexel University College of Medicine, Philadelphia, PA, 19102, USA

## Abstract

Studies in animal models show that the primary mechanism by which heme-oxygenases impart beneficial effects is due to the gaseous molecule carbon monoxide (CO). Produced in humans mainly by the catabolism of heme by heme-oxygenase, CO is a neurotransmitter important for multiple neurologic functions and affects several intracellular pathways as a regulatory molecule. Exogenous administration of inhaled CO or carbon monoxide releasing molecules (CORM’s) impart similar neurophysiological responses as the endogenous gas. Its’ involvement in important neuronal functions suggests that regulation of CO synthesis and biochemical properties may be clinically relevant to neuroprotection and the key may be a change in metabolic substrate from glucose to lactate. Currently, the drug is under development as a therapeutic agent and safety studies in humans evaluating the safety and tolerability of inhaled doses of CO show no clinically important abnormalities, effects, or changes over time in laboratory safety variables. As an important therapeutic option, inhaled CO has entered clinical trials and its clinical role as a neuroprotective and neurotherapeutic agent has been suggested. In this article, we review the neuroprotective effects of endogenous CO and discuss exogenous CO as a neuroprotective and neurotherapeutic agent.

## Introduction

CO is critical in the brain for a host of functions. Involvement of carbon monoxide (CO) in several aspects suggests that agents affecting the synthesis, transactions, and disposition of the gas have clinical relevance to neuroprotection [1,2]. Endogenous production originates from heme metabolism (at least 86%) and heme-independent sources which include auto- and enzymatic oxidation of phenols, photo-oxidation of organic compounds, iron-ascorbate-catalyzed lipid peroxidation of microsomal lipids and phospholipids, and reduction of cybochrome *b*_5_. Heme oxygenase isozymes catalyze the first and rate-limiting step in the degradation of heme to iron, biliverdin, and CO. The cytoprotective effects of the heme oxygenases are attributed to the production of CO. The majority of CO formed is taken up in the cytosol before being released and combined with hemoglobin and thus, correlation of carboxyhemoglobin (COHb) levels with biological changes induced by CO and remnant effects of CO after COHb elimination is poor. A new method for measuring the rate of endogenous CO production in humans described by Coburn and colleagues allows calculation of the rate of heme catabolism with a precision of ± 2 μmol/h and is applicable as a diagnostic and therapeutic tool in neurophysiology, neurometabolism, and neurologic pathologies [3].

## Production and neuroprotective effects of endogenous carbon monoxide

Endogenous formation of neuronal CO is dependent on the expression of brain heme oxygenases. Heme oxygenase-1 (HO-1) is primarily localized in endoplasmic reticulum, but has been isolated in cytoplasm, nuclear matrix, mitochondria, and peroxisomes. Under basal conditions, tissues not involved in red blood cell or hemoglobin metabolism have low to undetectable levels, but the enzyme is ubiquitously induced. Heme oxygenase-2 (HO-2) proteins are primarily anchored to the endoplasmic reticulum. These two isoforms have been extensively investigated. Hayashi and colleagues studied the structure of the third isoform (heme oxygenase-3) in the rat with genomic PRC and found two HO-3-related genes (HO-3a and HO-3b). The authors suggest that HO-3 is nonfunctional and that the HO-3a and HO-3b genes are processed pseudogenes derived from HO-2 transcripts [4]. Isoforms in the rat brain assessed by real-time quantitative RT-PCR are greatest for HO-2 seen throughout the brain at much higher levels than HO-1 and HO-3. The highest levels of expression are in the cerebellum and the hippocampus. HO-1 and HO-2 are detectable in both cortical neurons and type I astrocytes [5]. The isoform HO-1 is highly expressed in select neurons in the hilus of the dentate gyrus, hypothalamus, cerebellum, and brainstem whereas HO-2 is more widely expressed in mitral cells in the olfactory bulb, pyramidal cells in the cortex and hippocampus, granule cells in the dentate gyrus, and many neurons in the thalamus, hypothalamus, cerebellum and caudal brainstem [6]. Under normal conditions, the HO-2 constitutive isoform accounts for nearly all of brain heme oxygenase activity and therefore CO production.

Physiologic functions in the brain attributed to endogenous CO to date include regulation of the hypothalamic-pituitary-adrenal axis, circadian rhythm control, odor response adaptation, nociception and chemoreception regulation, hearing, long-term potentiation, neuroendocrine regulation, behavior modification, memory, and vision [7-10]. The function of HO-2 in the central nervous system have been defined using HO-2 gene deletion and pharmacological inhibitors/activators of the enzyme in animal models and cultured cells of neurons, astrocytes, and cerebral vascular endothelial cells [11-16]. Studies by Doré and colleagues showed that HO-2 deletion results in increased neurotoxicity in cultured brain cells and increased damage following transient cerebral ischemia in mice [17]. Several authors have shown that pharmacologic inhibition or gene deletion of brain HO-2 exacerbates oxidative stress induced by seizures, glutamate, and inflammatory cytokines, and causes cerebral vascular injury [18-22]. Exposure of cortical neurons to glutamate increases HO-2 activity and CO production by calcium-calmodulin in a calcium-dependent manner, a process that occurs in milliseconds [23]. Heme oxygenase-2 is also activated during neuronal stimulation by phosphorylation by CK2 and may be more long-term [24]. Stimulation of metabotropic and ionotropic glutamate receptors lead to increased CO production as well [25-27]. Brain homeostasis and neuronal survival during seizures, hypoxia and hypotension correspond to upregulation of HO-2 expression with CO production and resulting neuroprotection [28].

The inducible isoform HO-1 has been targeted for neuroprotection and neuroinflammation in several diseases. Evidence suggests that the pathogenesis of several neurodegenerative diseases including Parkinson’s disease, Alzheimer’s disease, Friedreich’s ataxia, multiple sclerosis, amyotrophic lateral sclerosis, and Huntington’s disease may be due to formation of reactive oxygen species and/or reactive nitrogen species with mitochondrial dysfunction [29-31]. HO-1 induction with resultant formation of CO is disrupted as is the protective system potentially active against brain oxidative injury and has been targeted for therapeutic interventions [32-36].

## Exogenous carbon monoxide as a neuroprotector

The beneficial effects of HO-1 and HO-2 have been attributed to CO. However neuroprotection using exogenous CO as inhaled carbon monoxide (CO) or injectable carbon monoxide releasing molecules (CORM) is a novel and underexplored strategy. Well known as a toxin at high doses, exogenous CO also has critical physiologic and cytoprotective properties at low concentrations and has known anti-apoptotic, anti-inflammatory, antiproliferative, and metabolic properties [37-41]. Studies by Vieira and colleagues showed that the preconditioning of murine primary cerebellar granule cells with exogenous CO prevented neuronal apoptosis induced by excitotoxicity and oxidative stress [42]. Zeynalov and colleagues evaluated the role of inhaled CO following 90-minutes of transient focal brain ischemia in a mouse model. Inhalation of 125 parts per million (ppm) or 250 ppm CO begun immediately at the onset of reperfusion resulted in reduction of hemispheric infarct volume, improved neurological deficit scores, and limited brain edema. Inhalation of 250 ppm CO begun 1 to 3 hours after ischemia resulted in reduction of infarct volume and improved neurological deficit scores [43]. Wang et al. exposed male wild-type and Nrf2-knockout mice to 250 ppm CO or control air for 18 hours immediately after permanent middle cerebral artery occlusion. Nrf2 is the principle transcription factor responsible for regulating HO-1 expression. CO neuroprotection was completely abolished in Nrf2-knockout mice suggesting that the beneficial effect of inhaled CO would at least partially be mediated through the Nrf2 pathway and therefore likely HO-1 [44]. In our recent report we found that piglets preconditioned with inhaled CO had less apoptosis in the neocortex/striatum and hippocampus after cardiopulmonary bypass (CPB) and deep hypothermic circulatory arrest (DHCA). Moreover animals treated with CO demonstrated a change in metabolic substrate utilization that correlated with neuroprotection [45].

## Exogenous carbon monoxide may change neurometabolism and result in neuroprotection

Modulation of neurometabolic pathways and resultant neuroprotection is likely dependent on the dose and timing of exogenous CO administration, either pre, peri, or post stressor. The challenge is to effect safe and effective CO concentrations in neural tissues without producing deleterious effects and to define the neural cellular targets and metabolic pathways. Recently, Queiroga and colleagues concluded that CO controls mitochondrial functioning, oxidative metabolism, and substrate utilization [46]. In the intact brain, interactions between neurons and astrocytes are requisite for brain energy metabolism, neuroprotection, and normal functions of the brain which are mainly excitation and conduction, electrical energy being derived from chemical processes through cerebral metabolism. Glucose has been considered the obligatory substrate for brain metabolism, but there has been continued debate as to the primary energy substrate of brain cells during basal and stressed conditions [47]. Distribution of energy substrates from the systemic circulation into neurons is principally determined by astrocytes and the dependence of cerebral function on blood glucose as a fuel does not exclude lactate or other substrates as an energy source. Lactate is used as a metabolic substrate by the brain, but the blood–brain transport of lactate is limited. Working brain tissue, however, releases large amounts of lactate. Uptake by carrier-mediated facilitated diffusion is limited by dependence on metabolism of accumulated lactate to maintain a concentration gradient. At physiologically occurring lactate concentrations, lactate uptake is at most 25% of the rate of glucose oxidation. In the classical model of brain metabolism, glucose can be utilized by both neurons and astrocytes through oxidative metabolism. With normoxia, glucose is readily consumed by both types of cells and lactate is produced by both neurons and astrocytes. Lactate leaves the astrocyte quickly, but little is transported out of the neuron. ATP produced within the mitochondria are similar between cells. However, ATP levels in the cytoplasm of neurons can be much higher than what is present in the cytoplasm of astrocytes. With hypoxia, glucose consumption does not change significantly between cells, however, lactate metabolism changes dramatically. Extracellular lactate rises and mitochondria ATP production is slightly reduced in both neurons and astrocytes. A second model of brain energy metabolism, the astrocyte-neuron lactate shuttle hypothesis, allows that astrocytes consume glucose through anaerobic glycolysis to pyruvate and then to lactate. The lactate is secreted into the extracellular space and can be taken up by neurons and used as a metabolic substrate. Exogenous CO may result in a change in metabolic substrate in the brain and may define the role of exogenous CO in neurometabolism and, subsequent, neuroprotection. This will be dependent as well on availability of specific metabolic substrates.

In a rat hippocampal slice preparation, Schurr and colleagues studied the combined effects of hypoxia and lactic acidosis on neuronal function. There were no significant decreases in recovery rate of synaptic function between control slices and experimental slices that were perfused with artifical cerebrospinal fluid containing 1.0, 2.0, 10.0, or 20.0 mM lactic acid 30 minutes before and during hypoxic insult. The authors concluded that neuronal tissue appears to be able to handle excess lactic acid [48]. Several studies show that lactate may be the preferred energy substrate of activated neurons and is neuroprotective [49-54] and lactate may be a major substrate for the mitochondrial tricarboxylic acid cycle. Lactate preserves neuronal function in experimental models of excitotoxicity, posthypoxic recovery, cerebral ischemia, and energy deprivation and can sustain neuronal integrity as an alternative energy substrate. In newborn piglets with intrauterine growth restriction (IUGR), Moxon-Lester et al. showed that during hypoxia brain lactate in some IUGR piglets were higher than in other IUGR piglets and normal weight piglets and that apoptosis in the frontal cortex and thalamus of IUGR piglets with high brain lactate were lower than IUGR piglets with low brain lactate. The authors concluded that increased brain lactate during hypoxia may be neuroprotective in IUGR piglets [55]. In a rat model of acute/severe hypoglycemia, Won and colleagues concluded that supplementation of glucose with lactate reduced neuronal death in the hippocampus and hypothesized that increasing brain lactate in this model offsets the decrease in NAD^+^ due to overactivation of PARP-1 by acting as an alternative energy substrate that can effectively bypass glycolysis and be fed directly to the citric acid cycle to maintain cellular ATP levels [56]. Our results in newborn piglets preconditioned with inhaled CO before CPB/DHCA are also consistent with a change to lactate as the metabolic substrate and resulting neuroprotection [45].

**Figure 1:**
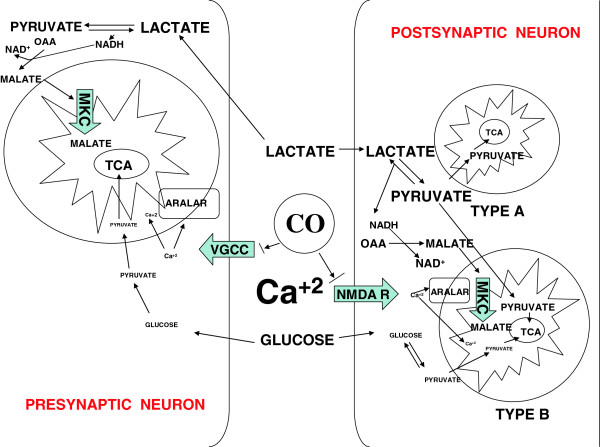
**Proposed mechanisms of effect of CO on presynaptic neurons and postsynaptic neurons on change to lactate as metabolic substrate.** Ca^2+^ signaling has been proposed as the primary determiner of change from glucose to lactate metabolism in the brain. CO could block both NMDA R and VGCC allowing less Ca^2+^ to reach the cytoplasm. This would decrease the amount of glucose available for the mitochondrial TCA cycle in both the presynaptic and postsynaptic neurons. Physiologic cystosol Ca^2+^ also binds to aralar which allows an increase in malate transfer into the mitochondria and a change to the CiMASH pathway.

Ca^2+^ signaling (which can be modulated by CO) may control the switch between glucose and lactate utilization during synaptic activity. (Figure 1) CO is able to regulate several classes of ion channels including calcium activated K(+), voltage-activaged K(+) and Ca(2+) channel families, ligand-gated P2X receptors, tandem P domain K(+) and channels and epithelial Na(+) channel. The calcium-activated potassium channels (BK(Ca)) are distributed in both excitable and non-excitable cells and are involved in action potential repolarization, neuronal excitability, neurotransmitter release, hormone section, tuning of cochlear hair cells, innate immunity, and modulation of smooth muscle tone. These channels are highly sensitive to intracellular calcium concentrations and voltage. Functionally, they are able to decrease voltage-dependent Ca(2+) entry through membrane hyperpolarization and serve as negative feedback regulators. The mechanisms by which CO regulates the calcium channels are unclear, remain controversial, and requires further study. However, Telezhkin and colleagues found that cysteine residue 911 in the C-terminal tail of human BK(Ca)α subunit is important for activation by CO [57]. In cultured mouse glutamatergic neurons, Bak et al. evaluated the effect of an ionomycin-induced increase in intracellular Ca^2+^ on glucose and lactate metabolism and concluded that glucose utilization is positively correlated with intracellular Ca^2+^ but that lactate utilization is not. The authors proposed a compartmentalized CiMASH (Ca^2+^-induced limitation of the malate-aspartate shuttle) that defines pre- and post-synaptic compartments metabolizing glucose and glucose plus lactate in which the latter displays a positive correlation between oxidative metabolism of glucose and Ca^2+^ signaling [58].

Almeida and colleagues studied the effects of CO exposure on primary cultures of astrocytes and showed that CO prevented apoptosis, increased ATP, improved oxidative metabolism, decreased lactate production, reduced glucose use, increased cytochrome c oxidase enzymatic specific activity, stimulated mitochondrial biogenesis and enhanced Bcl-2 expression [59]. Vieira and colleagues studied preconditioning of primary cultures of mice cerebellar granule neurons with CO to prevent apoptosis induced by excitotoxicity (induced by glutamate) and oxidative stress (induced by tert-butylhydroperoxide). Pretreatment with CO protected neurons from apoptosis in all ranges of glutamate concentrations tested and decreased neuronal apoptosis at 16 and 20 μM of the oxidant agent after CO exposure. The authors evaluated the preconditioning mechanism and propose that exogenous CO induces intracellular ROS generation which activates nitric oxide synthase (NOS) (increased NO production) that in turn activates sGC leading to increased levels of cGMP and the opening of ATP-dependent mitoK_ATP_ (important for neuronal cell protection) [42]. In vivo studies have not been done. Other proposed mechanisms for CO effects include mitochondrial biogenesis (more mitochondria are produced with the resulting increase in ATP production) and improved oxidative phosphorylation. CO effects on mitochondria may be due to partial and/or reverse inhibition of cytochrome c oxidase (complex IV) and/or accelerated oxidative phosphorylation resulting in ROS as important signalling molecules in the cell. CO inhibits loss of mitochondrial potential, and the opening of an 800 Da pore through the inner membrane, resulting in swelling and cytochrome *c* release.

## Clinical application of inhaled carbon monoxide for neuroprotection

Inhaled CO is an important therapeutic option and has entered clinical trials (http://www.clinicaltrials.gov). Studies in humans performed by INO Therapeutics LLC evaluated the safety and tolerability of inhaled single doses of carbon monoxide when administered as an inhaled gas for approximately 1 hour to healthy males (randomized, single blind, placebo controlled in parallel groups). Doses of 0.2, 0.75, 2.0, and 2.3 mg/kg/hr resulted in mean total maximum COHb levels of 2.0%, 3.4%, 7.7%, and 8.8%, respectively. All doses were well tolerated. Analyses of neurocognitive test data could not detect evidence of any acute or delayed differences in response between exposure to any of the carbon monoxide doses. The second study in humans performed by INO Therapeutics LLC was a randomized, single-blind study conducted in four panels of subjects. A total number of 12 healthy male volunteers received carbon monoxide or placebo by inhalation (ten subjects receiving CO and 2 subjects receiving placebo). In Panel 1, 12 subjects were given repeated doses of 2.3 mg CO/kg or placebo during 1 hour for 10 consecutive days. Panel 2 include 12 subjects receiving a single dose of 3.0 mg CO/kg or placebo. Panel 3 included 12 subjects given repeated doses of 3.0 mg CO/kg or placebo during 1 hour for 10 consecutive days. Panel 4 was a crossover study that included 12 subjects receiving a 3.0 mg CO/kg single dose sourced from a 5.97 mg/L drug product and a 3.0 mg CO/kg single dose sourced from a 12 mg/L drug product. The highest level of COHb measured was 13.9% in the 3.0 mg/kg/hour dose. These studies indicate that inhaled CO is safe and tolerable in humans. Clinical trials using inhaled CO include Carbon Monoxide Therapy for Severe Pulmonary Arterial Hypertension, Study of Inhaling Carbon Monoxide to Treat Patients with Intestinal Paralysis after Colon Surgery, Study of Inhaled Carbon Monoxide to Treat Idiopathic Pulmonary Fibrosis, Modification of Chronic Inflammation by Inhaled Carbon Monoxide in Patients with Stable Chronic Obstructive Pulmonary Disease (COPD), and Carbon Monoxide to Prevent Lung Injury. Clinical application in neurological disorders remains unexplored however anecdotal data suggest that smokers have a very low incidence of Alzheimers disease [60].

Preclinical studies of protective conditioning, a powerful laboratory strategy used to evaluate metabolic pathways and cell death, using many different stimuli show less pathology in models of epilepsy, stroke, hypoxia-ischemia, traumatic brain injury, and craniocerebral tumor resection [61-71]. Clinical application of inhaled CO as a neuroprotective agent (the agent does not have to be the same as the potentially lethal insult) could be as a preconditioning agent, postconditioning agent, and/or periconditioning agent and may benefit patients undergoing cardiopulmonary bypass for heart surgery, extracorporeal membrane oxygenation, resection of brain tumors/abscesses or vascular malformations, deep hypothermic circulatory arrest, radiation or chemotherapy for brain tumors, traumatic brain injury, hypoxic injury of the newborn, stroke, epilepsy, and neurodegenerative diseases. Timing and dosing for maximum effect and safety needs to be evaluated in clinical trials for these indications.

## Conclusion

The role of CO in the brain and central nervous system (CNS) has historically been negative, however recent data as presented in this review suggests that this dogma needs to be reevaluated. Endogenous CO is critical for normal brain function. Clearly CO is produced in the brain as a neurotransmitter and regulates memory and circadian rhythms. Therefore how can it also be so potently toxic. The answer is, of course, the dose and duration of exposure. The reports of the beneficial effects of CO in the brain and CNS continue to emerge and serve as a call for this simple gas to be reevaluated. Indeed, CO is currently being tested in clinical trials after passing rigorous safety testing. CO, like NO before it, may prove to be a therapeutic option and a new and novel approach to various neuropathologies. Clearly the time has come to reassess this simple gas as one cannot ignore the remarkable data that continues to be reported. The role for CO as a neurotherapeutic based on compelling animal data necessitates further testing in humans.

## Competing interests

The author has no competing interests.
